# Analysis of Different Positions of Fiber-Reinforced Composite Retainers versus Multistrand Wire Retainers Using the Finite Element Method

**DOI:** 10.1155/2014/581029

**Published:** 2014-10-22

**Authors:** Arezoo Jahanbin, Mostafa Abtahi, Farzin Heravi, Mohsen Hoseini, Hooman Shafaee

**Affiliations:** ^1^Dental Research Center and Department of Orthodontics, School of Dentistry, Mashhad University of Medical Sciences, Mashhad 9177948959, Iran; ^2^Dental Materials Research Center and Department of Orthodontics, School of Dentistry, Mashhad University of Medical Sciences, Mashhad 9177948959, Iran; ^3^Dental Research Center and Department of Orthodontics, School of Dentistry, Birjand University of Medical Sciences, Birjand, Iran; ^4^Department of Orthodontics, Dental School, Park Square, Mashhad 9177948959, Iran

## Abstract

*Background.* The aim of this study was to evaluate root displacement of the lower incisors fixed with FRC in different positions versus FSW retainers using the finite element method. *Materials and Methods.* 3D finite element models were designed for a mandibular anterior segment: Model 1: flexible spiral wire bonded to the lingual teeth surfaces, Model 2: FRC bonded to the upper third of lingual teeth surfaces, and Model 3: FRC bonded to the middle third. FE analysis was performed for three models and then tooth displacements were evaluated. *Results.* In contrast to lateral incisors and canines, the FSW retainer caused the central teeth to move more than the teeth bonded with FRC in both loadings. Comparison between Models 2 and 3 (in vertical loading) showed that FRC retainers that bonded at the upper third of lingual teeth surfaces made central and canine teeth move less than FRC retainers bonded at the middle third; however, for lateral teeth it was the opposite. *Conclusion.* FRC retainers bonded at the upper third of lingual teeth surfaces make central and canine teeth move less than FRC retainers bonded at the middle third in vertical loading; however, for lateral teeth it was the opposite.

## 1. Introduction

Contemporary retaining strategies in orthodontics basically include removable and fixed retainers. Fixed retainers are used principally for long-term retention of treated orthodontic cases and for the permanent splinting of periodontally involved teeth [1]. Moreover, they have significant advantages for patient comfort and esthetic acceptability [2].

Two different forms of fixed retainers are widely used in orthodontics: multistrand wire retainers and fiber-reinforced composite retainers. The main advantage of the use of multistrand wires is the irregular surface that offers increased mechanical retention for the composite without the need for the placement of retentive loops [3]. Moreover, another asset is the flexibility of the wire that allows physiologic movement of the teeth, even when several adjacent teeth are bonded [4].

Limitations, however, include aesthetics and the fact that they cannot be used in patients with a nickel allergy. Therefore, alternatives have been developed such as fiber-reinforced composite retainers. Fiber-reinforced composite (FRC) containing various fibers such as carbon, polyaramid, polyethylene, and glass has received increasing acceptance as restorative materials [5]. Reinforced polyethylene fiber material was successfully used for fixed orthodontic retainers [6]. It can adapt easily to dental contours and be manipulated during the bonding process. It also has acceptable strength because of the integration of fibers with composite resin that leads to good clinical longevity [7]. Moreover, it can also connect closer to the incisal edges of teeth, which are useful from biological and biomechanical perspectives.

Although FRC bonded to enamel has acceptable bond strength, further information is needed on the behavior of FRC lingual retainers in different positions under occlusal forces [5].

As finite element analysis (FEA) is a useful method for analyzing the interaction between materials and forces and the pattern of stress distribution in a given mass. The aim of this study was to evaluate stress distribution in the PDL of lower incisors fixed with FRC in different positions versus multistrand wire retainers using the finite element method.

## 2. Materials and Methods

In this study, the anterior segment of the mandibular dental arch with six incisor teeth (canine to canine) was modeled.

In the first step, two-dimensional pictures of lower six anterior teeth were obtained and then the captured data were plotted using a 3D CAD design software (Mechanical Desktop R2.0, Autodesk Inc., CA, USA) to construct a 3D solid model, which was then imported into an FE analysis software (ANSYS 5.4, ANSYS Inc., Canonsburg, PA, USA).

The object to be studied is graphically simulated in a computer in the form of a mesh that defines its geometry. In a process called discretization, this mesh is divided into a number of subunits termed elements, which are connected at a finite number of points called nodes. The size of the elements determines the accuracy of the calculations; therefore, each model had approximately 119000 elements with 0.2 mm dimension. The type of elements was SOLID 92 and the final element on the *x*-, *y*-, and *z* axes of the model base was assumed to be fixed, thereby defining the boundary conditions. No adhesive layer was created in this study, because complete bonding with a very thin adhesive layer would not cause any difference in stress distribution results by FE analysis [8].

All the elements contributing to the model were assumed to be homogenous. The mechanical properties assigned to the elements were linear-elastic and Poisson's ratio was considered at 0.3 for all of them except PDL, which was considered 0.45. Three different Young's moduli were chosen to represent cortical bone (13.7 GPa), sponge bone (1.37 GPa), PDL (0.68 × 10^−3^ GPa), and tooth structure (18.6 GPa). The PDL width was considered 0.5 mm. The properties of FRC and multistrand wire used in this study for FE analysis are listed in Table 1.

**Figure 1:**
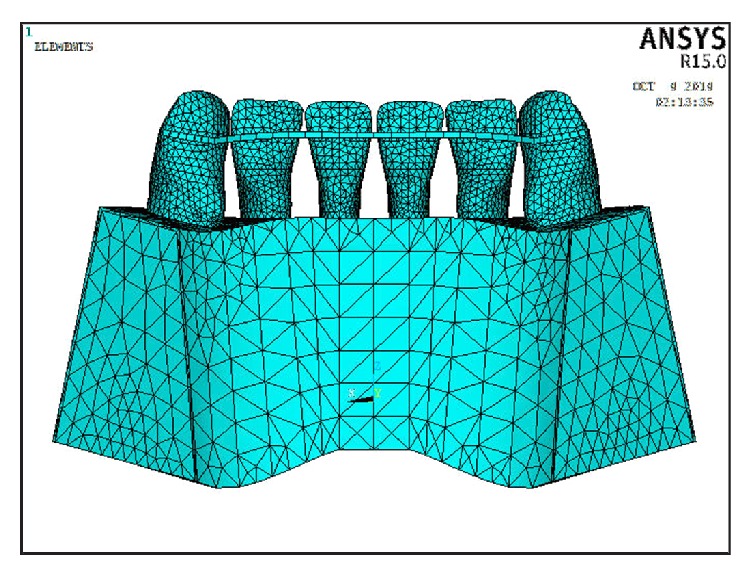
Model 1 in which the flexible spiral wire bonded to the lingual teeth surfaces.

**Figure 2:**
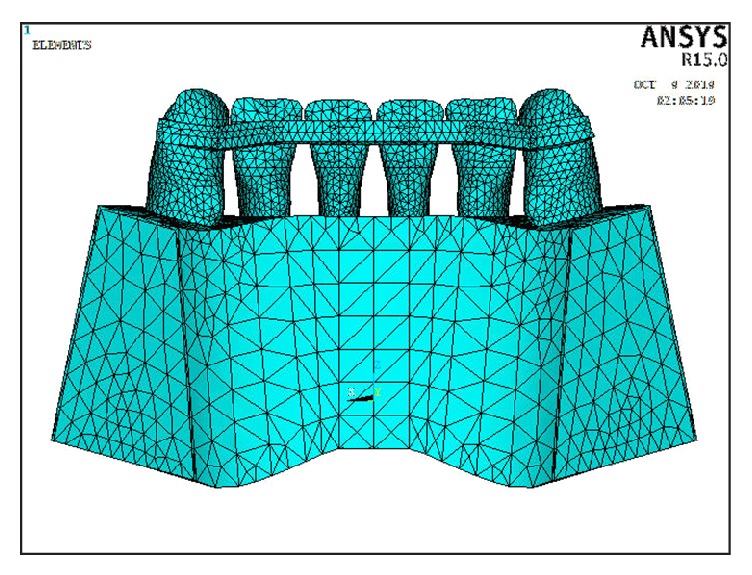
Model 2 in which FRC bonded to the upper third of lingual teeth surfaces.

**Figure 3:**
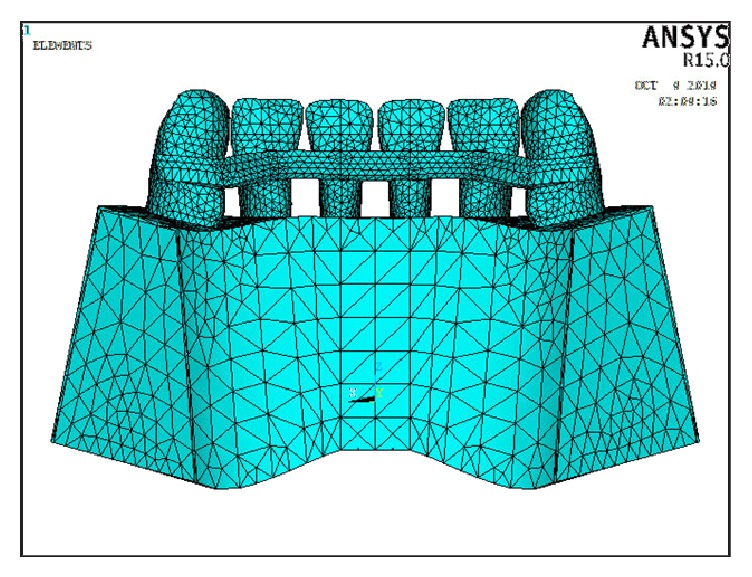
Model 3 in which FRC bonded to the middle third of lingual teeth surfaces.

In this study, three models were constructed:

 In Model 1, six lower incisor teeth were connected together via a continuous multistrand wire that was attached from the distal marginal ridge of the canine on one side to the distal marginal ridge of the canine on the opposite side on the middle third of the teeth.
 The multistrand wire (19.5 mm equal to 0.5 mm in width) was placed 6 mm gingival to the incisal edge in such a way to form a circular arc with these dimensions (Figure 1).
 In Model 2, six lower incisor teeth were connected together via a continuous bar of FRC that was attached from the distal marginal ridge of the canine on one side to the distal marginal ridge of the canine on the opposite side, 5 mm gingival to the incisal edge on the middle third of the teeth (Figure 2). To examine the mechanical behavior of the FRC bar, unidirectional glass fibers (Everstick Ortho, Stick Tech LTD, Turku, Finland) were used.
 In Model 3, six lower incisor teeth were connected together via a continuous bar of FRC that was attached from the distal marginal ridge of the canine on one side to the distal marginal ridge of the canine on the opposite side, 2 mm gingival to the incisal edge on the upper third of the teeth (Figure 3).

The mentioned FRC (0.75 mm in thickness and 2.1 mm in width) was placed in such a way to form a circular arc with these dimensions in Models 2 and 3.

In this study, two loading conditions were tested on each FE model: a vertical load on the incisal edge of the midline to simulate vertical loading while biting and lingual loading from the labial surface of the teeth to simulate protrusive jaw movement during mastication.

For vertical loading, a vertical load of 10 N was applied to the midline to simulate the maximum biting force. To simulate lingual loading during protrusive jaw movement, a 10 N force was applied to the labial surface of the two central incisors at an angle of 90 degrees from the labial direction.

FE analysis was presumed to be linear static. FE model construction and FE analysis were then performed for the three models using the finite element analysis software ANSYS 5.4.

## 3. Results

Displacements of central and lateral incisors and canine were measured in six locations (apex palatal, apex labial, middle root palatal, cervix labial, and cervix palatal) after vertical loading and protrusive loading in all three models.

In Model 1, the maximum displacement of the central incisors occurred at the apex palatal and apex labial in vertical loading and protrusive loading, respectively. The minimum displacement was found at the middle root labial and middle root palatal in vertical loading and protrusive loading, respectively.

For the lateral incisor and canine, maximum displacement was recorded at the apex palatal in both vertical loading and protrusive loading and the minimum displacement was found at the middle root labial of the lateral in both types of loading, while the minimum displacement in the canine was found at the middle root labial and apex labial in vertical loading and protrusive loading, respectively (Table 2).

In Model 2, the maximum displacement of central incisors was recorded at the apex labial in both vertical loading and protrusive loading. The minimum displacement of the central incisor was observed at the middle root palatal in vertical loading and at the cervix labial and cervix palatal in protrusive loading. The maximum displacement of the lateral incisor was recorded at the apex labial in vertical loading and at the apex palatal in the protrusive loading. The minimum displacement of the lateral incisor was observed at the apex palatal and middle root palatal in vertical loading and at the middle root palatal in protrusive loading. The canine showed maximum displacement at the apex labial in both types of loading. The minimum displacement of the canine was observed at the middle root labial in vertical loading and at the middle root palatal in protrusive loading (Table 2).

Displacement data of Model 3 shows that the maximum displacement of the central incisor occurred at the apex labial in both vertical loading and protrusive loading. Minimum displacement was observed at the middle root palatal in vertical loading and at the middle root labial in protrusive loading. The maximum displacement of the lateral incisors was recorded at the apex palatal in both types of loading. The minimum displacement of the lateral incisor was observed at the middle root labial in both types of loading. Canine showed maximum displacement at the apex labial in vertical loading and at the apex palatal in protrusive loading. The minimum displacement of the canine was observed at the middle root palatal in vertical loading and at the apex labial in protrusive loading (Table 2).

Table 3 shows the displacement proportion in vertical loading and protrusive loading among the groups.

M1/M2 proportion for central teeth showed that, in vertical loadings, multistrand wires cause more movement than FRC retainers bonded at the upper third of lingual teeth surfaces; however, for lateral and canine teeth, it was the opposite and multistrand wire made the teeth move less than FRC.

M1/M3 proportions for central teeth showed that, in both types of loading, the teeth with multistrand wires moved more than FRC retainers bonded at the middle third of the lingual teeth surface. For lateral and canine teeth, it was opposite and multistrand wire made the teeth less move than FRC.

M2/M3 proportions for the central incisors showed that, in vertical loading, the teeth with FRC retainers bonded at the upper third of the lingual teeth surfaces moved less than FRC retainers bonded at the middle third. The results were the same for the canine; however, for lateral teeth, it was the opposite.

In protrusive loading, the central incisors with FRC retainers bonded at the upper third moved more than FRC retainers bonded at middle third, except at the cervix labial and cervix palatal. For lateral incisors, M2/M3 data showed that the displacements of the teeth with FRC retainer bonded at the upper third of the lingual surface were less than the teeth with FRC retainer bonded at the middle third. For canine incisors, displacements of the teeth were more when FRC bonded to the upper lingual third, except at the apex palatal and middle root palatal.

## 4. Discussion

Many studies have evaluated the effects of various wire types and sizes in fixed retainers and recently fiber-reinforced materials have been used widely [9, 10].

Fixed retainers are used principally for long-term retention and they need no patient compliance. In the recent decade, orthodontists have become well aware of the benefit of fixed retainers in relapse control [11]. The main question at this phase is their biomechanical efficacy. Geramy et al. evaluated anterior teeth splinting after orthodontic treatment using FEM. Two sizes of wire (0.016 inch) and (0.016 × 0.022 inch) were used in this study. They concluded splint cases (with round or rectangular wires) can benefit from stress redistribution when biting small food particles and in lateral movements [12].

FRC retainers are more esthetic than FSW retainers. Moreover, they can also connect closer to the incisal edges of teeth, which are useful from the biological and biomechanical standpoint [7]. In our study, stress distribution in the PDL of lower incisors fixed with FRC in different positions versus multistrand wire retainers was evaluated using FEM. In all three models, the maximum displacement of central and lateral incisors and canines was observed more frequently at the apex palatal and then at the apex labial in both vertical loading and protrusive loading; therefore, maximum stress was located at the apex. Minimum displacement was often recorded at the middle root labial and middle root palatal; therefore, stress was minimal at the middle root in all three groups.

From a biomechanical standpoint, bonding FRCs to different locations of the lingual teeth surfaces and bonding multistrand wire to the middle third of lingual teeth have different stress distribution patterns. The displacement ratios among the three models are shown in Table 3. These ratios determined the location of stress concentration when the models were compared to each other and so it can be determined which location will have the lowest stress concentration in vertical and protrusive loading.

The results of this study show that the effects of types of bonded retainer on tooth displacement vary for the central, lateral, and canine teeth. Also, type of loading may cause differences. On the other hand, in mandibular movements, splinting teeth together with bonded retainers may have certain disadvantages. When the teeth bonded with FSW retainer, central teeth move more in comparison to teeth bonded with FRC. However, multistrand wire makes lateral and canine teeth move less. These findings were the same for both types of loading. Comparison between Models 2 and 3 (in vertical loading) showed that FRC retainers bonded at the upper third of the lingual teeth surfaces make central and canine teeth move less than FRC retainers bonded at the middle third; however, for lateral teeth, it was the opposite. In protrusive loading, FRC retainers bonded at the upper third of the lingual teeth surfaces make central and canine teeth move more than FRC retainers bonded at the middle third; however, for lateral teeth, it was the opposite.

## 5. Conclusion

According to the results of this FEM study, in all three models, the maximum displacement of central, lateral, and canine teeth was observed more frequently at the apex palatal and then at the apex. Minimum displacement was observed at the labial in both vertical loading and protrusive loading; therefore, the maximum stress was located at the apex. In addition, FSW retainers caused more movement in the central root than FRC bonded retainers in vertical and protrusive loadings. However, multistrand wire makes lateral and canine teeth move less in both loadings. In addition, FRC retainers bonded at the upper third of lingual teeth surfaces made central and canine teeth move less than FRC retainers bonded at the middle third in vertical loading; however, for lateral teeth, it was the opposite.

## Figures and Tables

**Table 1:** Material properties used in this study.

Material	Young's modulus (GPa)	Poisson's ratio	Shear Modulus (GPa)
FRC longitudinal (*X*)	46	0.39	16.5
Transverse (*Y*)	7	0.29	2.7
Transverse (*Z*)	7	0.29	2.7
Multistrand wire	90	0.3	34.6

**Table 2:** Model 1 to 3 displacement data (in millimeters).

Tooth	Direction	Vertical loading			Protrusive loading		
		Model 1	Model 2	Model 3	Model 1	Model 2	Model 3
Central	Apex labial	0.2655	0.1750	0.2160	0.8994	0.5525	0.4437
	Apex palatal	0.2669	0.1487	0.1736	0.8366	0.4742	0.3310
	Middle root labial	0.2589	0.1690	0.2066	0.3999	0.3386	0.2659
	Middle root palatal	0.2668	0.1474	0.1724	0.3391	0.2531	0.0853
	Cervix labial	0.2596	0.1593	0.1942	0.6486	0.3058	0.3159
	Cervix palatal	0.2596	0.1593	0.1942	0.6486	0.3058	0.3159

Lateral	Apex labial	0.0065	0.0734	0.0585	0.0395	0.1253	0.1571
	Apex palatal	0.0092	0.0651	0.0612	0.1048	0.1433	0.2088
	Middle Root labial	0.0064	0.0713	0.0562	0.0185	0.0634	0.0854
	Middle Root palatal	0.0088	0.0651	0.0610	0.0412	0.0515	0.1153
	Cervix labial	0.0085	0.0676	0.0576	0.0571	0.0986	0.1095
	Cervix palatal	0.0085	0.0676	0.0576	0.0571	0.0986	0.1095

Canine	Apex labial	0.0016	0.0183	0.0263	0.0230	0.0906	0.0553
	Apex palatal	0.0022	0.0215	0.0226	0.0853	0.1531	0.1785
	Middle Root labial	0.0008	0.0061	0.0110	0.0292	0.0820	0.0808
	Middle Root palatal	0.0017	0.0061	0.0108	0.0360	0.0773	0.1080
	Cervix labial	0.0015	0.0183	0.0192	0.0395	0.1000	0.0866
	Cervix palatal	0.0015	0.0183	0.0192	0.0395	0.1000	0.0866

**Table 3:** Displacement proportion in vertical loading and protrusive loading among the groups.

Tooth	Direction	Displacement proportion in vertical loading			Displacement proportion in protrusive loading		
		M1/M2	M1/M3	M2/M3	M1/M2	M1/M3	M2/M3
Central	Apex labial	1.5	1.2	0.8	1.6	2.0	1.2
	Apex palatal	1.7	1.5	0.8	1.7	2.0	1.4
	Middle root labial	1.5	1.2	0.8	1.1	1.5	1.2
	Middle root palatal	1.8	1.5	0.8	1.3	3.9	2.9
	Cervix labial	1.6	1.3	0.8	2.1	2.0	0.9
	Cervix palatal	1.6	1.3	0.8	2.1	2.0	0.9

Lateral	Apex labial	0.08	0.1	1.2	0.3	0.2	0.7
	Apex palatal	0.1	0.1	1.0	0.7	0.5	0.6
	Middle root labial	0.08	0.1	1.2	0.2	0.2	0.7
	Middle root palatal	0.1	0.1	1.0	0.8	0.3	0.4
	Cervix labial	0.1	0.1	1.1	0.5	0.5	0.9
	Cervix palatal	0.1	0.1	1.1	0.5	0.5	0.9

Canine	Apex labial	0.08	0.06	0.6	0.2	0.4	1.6
	Apex palatal	0.1	0.09	0.9	0.5	0.4	0.8
	Middle root labial	0.01	0.07	0.5	0.3	0.3	1.0
	Middle root palatal	0.1	0.15	0.5	0.4	0.3	0.7
	Cervix labial	0.08	0.07	0.9	0.3	0.4	1.1
	Cervix palatal	0.08	0.07	0.9	0.3	0.4	1.1

## References

[1] Lang G., Alfter G., Göz G., Lang G. H. (2002). Retention and stability—taking various treatment parameters into account. *Journal of Orofacial Orthopedics*.

[2] Scribante A., Sfondrini M. F., Broggini S., D'Allocco M., Gandini P. (2011). Efficacy of esthetic retainers: clinical comparison between multistranded wires and direct-bond glass fiber-reinforced composite splints. *International Journal of Dentistry*.

[3] Zachrisson B. U. (1982). The bonded lingual retainer and multiple spacing of anterior teeth. *Swedish Dental Journal*.

[4] Årtun J. (1984). Caries and periodontal reactions associated with long-term use of different types of bonded lingual retainers. *American Journal of Orthodontics*.

[5] Vallittu P. K. (1999). Flexural properties of acrylic resin polymers reinforced with unidirectional and woven glass fibers. *The Journal of Prosthetic Dentistry*.

[6] Rose E., Frucht S., Jonas I. E. (2002). Clinical comparison of a multistranded wire and a direct-bonded polyethylene ribbon-reinforced resin composite used for lingual retention. *Quintessence International*.

[7] Karaman A. I., Kir N., Belli S. (2002). Four applications of reinforced polyethylene fiber material in orthodontic practice. *American Journal of Orthodontics and Dentofacial Orthopedics*.

[8] Shinya A., Yokoyama D., Lassila L. V., Vallittu P. K. (2008). Three-dimensional finite element analysis of metal and FRC adhesive fixed dental prostheses. *The Journal of Adhesive Dentistry*.

[9] Artun J., Zachrisson B. (1982). Improving the handling properties of a composite resin for direct bonding. *The American Journal of Orthodontics*.

[10] Geserick M., Ball J., Wichelhaus A. (2004). Bonding fiber-reinforced lingual retainers with color-reactivating flowable composite. *Journal of Clinical Orthodontics*.

[11] Renkema A.-M., Al-Assad S., Bronkhorst E., Weindel S., Katsaros C., Lisson J. A. (2008). Effectiveness of lingual retainers bonded to the canines in preventing mandibular incisor relapse. *The American Journal of Orthodontics and Dentofacial Orthopedics*.

[12] Geramy A., Retrouvey J. M., Sobuti F., Salehi H. (2012). Anterior teeth splinting after orthodontic treatment: 3D analysis using finite element method. *Journal of Dentistry*.

